# Bentonite in Korea: A Resource and Research Focus for Biomedical and Cosmetic Industries

**DOI:** 10.3390/ma17091982

**Published:** 2024-04-24

**Authors:** Md Shohel Rana, Shukho Kim

**Affiliations:** 1 Department of Biomedical Sciences, The Graduate School, Kyungpook National University, Daegu 41944, Republic of Korea; msranabau5@gmail.com; 2 Department of Microbiology, School of Medicine, Kyungpook National University, Daegu 41944, Republic of Korea

**Keywords:** bentonite, biomedicine, cosmetics, antimicrobial

## Abstract

This study provides an in-depth review of bentonite, focusing on its applications in Korea’s biomedical and cosmetic sectors. It delves into bentonite’s chemical properties, which make it a valuable resource in various industries, particularly in the health and beauty industries. We discuss bentonite’s antimicrobial properties, showcasing its effectiveness against a wide range of pathogens and its potential as a biomedicine adjuvant to boost immune responses. Despite its benefits, the review also addresses the need for caution due to its possible side effects when used in human therapy. In the cosmetics industry, bentonite is prized for its ability to absorb impurities, making it a popular ingredient in products from leading brands. The review highlights the ongoing research and development efforts aiming to further explore bentonite’s capabilities and applications, underlining the material’s significant contribution to advancing Korea’s innovation in the biomedical and cosmetic fields. This review suggests that with more research, bentonite’s full potential can be unlocked, offering new opportunities for these industries.

## 1. Introduction

Bentonites are clay rocks formed from volcanic glass and primarily consist of montmorillonite (a type of smectite), which is a hydrous aluminous sheet silicate of the exchangeable interlayer cations, mostly calcium and sodium [1]. It is named after the town of Benton, Wyoming, where it was first discovered in 1890 [2]. Bentonites are used industrially due to their significant physical and chemical properties, including their crystal structures, mineralogical compositions, small particle sizes, large specific surface areas, high capacity for ion exchange, cation exchange capacity, swelling index, colloidal properties, dehydration, and reactions with organic and inorganic reagents [3]. These unique properties make bentonite useful in a variety of applications, including oil and gas drilling, construction [4], agriculture [5], environmental remediation [6], radioactive waste repositories [7], and pharmaceuticals and cosmetics [8]. Notably, its utilization has extended to the production of nanocomposites, representing a cutting-edge application in recent times [1]. However, the biomedical uses of natural clays pertain to their functions as excipients and/or active ingredients [9]. Natural clays are commonly used as active ingredients in gastrointestinal tract, anti-inflammatory, antidiarrheal, and dermatological treatments [10]. They also have several applications as active excipients, such as disintegrants, lubricants, anticaking agents, and emulsifying agents [9,10]. Furthermore, clay minerals are often used as drug carriers and as vehicles for the controlled release of therapeutic agents [9]. Additionally, bentonite is used in the personal care industry as a component in face masks, skin care products, and cosmetics due to its absorbent and detoxifying properties [11].

Bentonite is widely used in various bio-industries in the USA. Primarily, it serves as a natural and safe detoxification tool, effectively absorbing toxins from the body [12]. In the animal feed industry, bentonite enhances nutrient absorption in livestock, promoting better health and growth [13]. Additionally, it acts as a feed additive, reducing harmful bacteria in animal guts [14]. Animals sometimes eat clay materials to intake essential minerals and detoxifying bodies, through ion exchange, particularly after consuming toxic substances [15,16]. So, those animals are good at pelotherapy, which is a therapeutic technique that involves the application of natural mud, clay, or peat to the body to treat various health conditions. People in Pohang, South Korea, used to ingest bentonite, what they call ‘dduckdoll’, meaning ‘cake stone’, to treat diarrheal diseases for a long time [17]. Liquid bentonite was also used for centuries in China, usually in summer, for the treatment of occasional diarrhea [17,18].

Bentonite has been registered as fit for use in medicine, cosmetics, food additives, animal feed, and veterinary medicine in Korean Pharmacopoeia, cosmetic regulations, Food Code, Feed management Law, and Korean Laws and Guidelines on Veterinary Drug regulation, respectively. The Korea Institute of Geoscience and Mineral Resources (KIGAM) has been evaluating the potential of bentonite development for medical and pharmaceutical usages since 2017 in South Korea. There are big mines in Pohang and Gyungju, areas harboring high-quality bentonite and montmorillonite [19]. The KIGAM constructed a GMP (good manufacturing practices)-grade facility which can produce clay material medicine with quality control and clean manufacturing in 2019 in order to fulfill the requirements of the Korean Pharmacopoeia [20]. In accordance with the Korean Pharmacopoeia, functional clays, including bentonite, are tested for their properties, purity, and microbial limits, as well as their heavy metals, arsenic, and foreign matter measurements [21]. For metal impurities, the International Council on Harmonisation of Technical Requirements for Human Use (ICH) provides guidelines for the quality control of bentonite used for medicinal purposes [22]. It has been able to develop pharmaceutical quality bentonite, which South Korea had been fully imported from abroad until now, and supply it to related companies. 

This review emphasizes the key role of bentonite in Korea as a valuable resource and suggests future research directions in the biomedical and cosmetic industries primarily based on contemporary research results.

**Figure 1:**
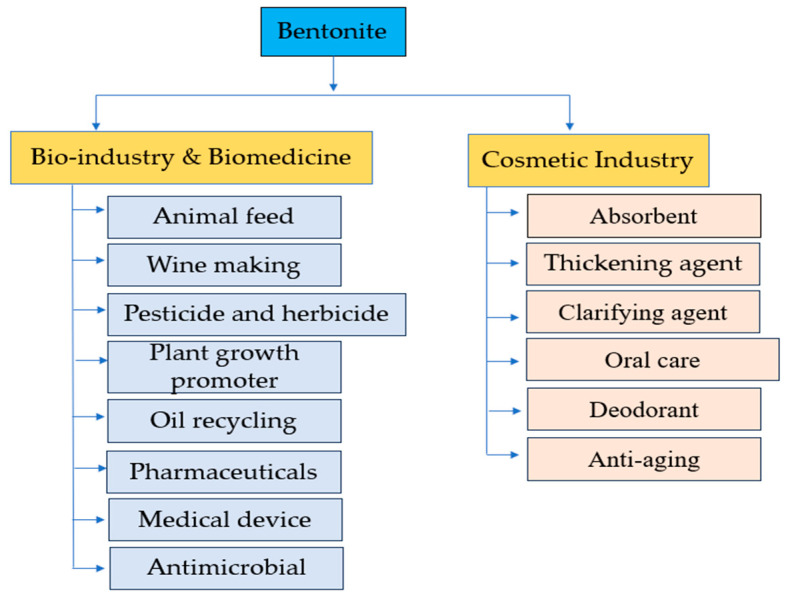
Summary diagram of bentonite applications in bio-industries and cosmetics industry.

The primary applications of bentonite clay in the biomedicine and cosmetic industries are described in this review; they are summarized in Figure 1.

## 2. Chemical Nature of Bentonite

Bentonite is chiefly composed of montmorillonite, a clay mineral belonging to the smectite group, featuring a structural arrangement where gibbsite layers interleave between silica layers, forming the fundamental structural unit [23,24]. The substitutions are primarily concentrated within the octahedral layer with ions like Mg^2+^ and Fe^2+^ and, to a lesser extent, between the silicate layer (Al^3+^/Si^4+^). The clay’s composition primarily revolves around the hydroxyl-aluminosilicate structure, with the crystalline framework being established by the interaction between tetrahedral silica layers and alumino-octahedral sheets. Within this framework, the partial substitution of Al^3+^ cations with ions like Mg^2+^ or Fe^2+^ occurs, followed by the incorporation of metals such as Na, K, Mg, or Ca to maintain charge balance [24]. This arrangement gives montmorillonite its high cation exchange capacity and swelling properties [25]. In addition, bentonite may contain various impurities, such as quartz, feldspar, and other minerals, depending on its source [26].

## 3. Bentonite Applications in Bio-Industries and Adjuvant Properties in Biomedicine

Bentonite, a versatile clay mineral, has many applications in bio-industries due to its unique properties. This section will address the various applications of bentonite clay in bio-industries.

*Animal feed*: Bentonite is used as a binder in animal feed and can help improve binding capacity, reduce caking and crusting, and increase nutrient absorption. It can also help to prevent digestive disorders and improve the overall health of cattle [26,27].

*Wine making*: Bentonite can be added to grapes (juice and skin) during fermentation. This helps to remove proteins and other unwanted substances, clean the mixture, and remove any remaining sediment. Additionally, it helps to reduce excess tannins, which can make wines bitter and sour [28].

*Environmental remediation*: Bentonite can be used to treat contaminated soil and groundwater due to its ability to absorb and remove contaminants such as heavy metals and organic pollutants [29].

*Pesticide and herbicide carrier*: Bentonite is used as a carrier for insecticides and herbicides so that the active ingredients are evenly distributed to the target area [30].

*Pharmaceuticals*: Bentonite is used as an excipient in the pharmaceutical industry, as it has absorbent and retentive properties and acts as a filler [31]. Bentonite also can be used as a binder in tablet formulations to hold the active ingredient and other excipients together [32]. In addition, bentonite plays roles in pharmaceutical applications:

- As a suspending agent: It keeps insoluble particles suspended in liquid formulations. Biophammer Co. in South Korea developed a bentonite–sorafenib complex that improved the solubility of an oral anticancer drug, sorafenib. The sorafenib molecules were encapsulated in the molecular state within the bentonite structure. Biobetters are aiming to develop an alternative drug delivery system (DDS) with bentonite [33].
- As a disintegrant: Bentonite can be used as a disintegrant to help break down a tablet or capsule and release the active ingredient [34]. The gel-like structure of bentonite allows it to retain drugs and vaccines in a controlled manner, releasing them gradually over time and improving their bioavailability. It is possible to develop a release-type formulation that enables the controlled release of the drug [35]. By adsorbing the drug to bentonite, the sustained release of the drug and long-term drug effects can be achieved. In one particular study on bentonite, most of the water-soluble drugs showed significantly higher adsorption rates over acidic to neutral hydrogen ion concentrations and significantly lower release rates than existing drugs in dissolution tests. With these features, it is possible to develop sustained-release formulations, for example, metformin with bentonite [36]. In addition to its drug delivery properties, bentonite has also been shown to enhance the immune response. Sodium bentonite was shown to improve the immunity of stinging catfish against *Aeromonas hydrophila* when it was included in their diet as a supplement/feed additive [37].
- As a thickener: Bentonite can be used as a thickener in ointments, creams, and lotions to give them a smooth, spreadable texture [38].
- As a clarifying agent: Bentonite can be used as a clarifying agent in the production of liquids such as syrups, suspensions, and emulsions to remove impurities and improve clarity [39].

*Nasal spray*: Bentonite is a type of clay mineral that contains a thin layer of aluminum silicate that has a negative charge; these qualities contribute to its capacity to adsorb viral particles and substances like drugs [40]. Nasal sprays containing bentonite can protect against SARS-CoV-2 and other airborne pathogens. The thixotropic nature of bentonite suspensions indicates that they exhibit reversible changes from a gel-like state when undisturbed to a liquid colloid upon agitation [41].

Further studies are also needed to assess the feasibility of its use in clinical applications and determine its long-term effects.

### 3.1. Antimicrobial Activity of Bentonite

Bentonite has been extensively studied for its antibacterial properties. It has been shown to exhibit antimicrobial activity against harmful microorganisms, including bacteria, virus, and fungi [42,43]. The exact mechanisms of this process are not fully understood, but it is believed to be due to the adsorption of bacteria and viruses on the surface of the bentonite particles, leading to their inactivation [44,45]. The antimicrobial effect of bentonite is attributed to its ability to absorb and bind bacterial cells, thus inhibiting their growth and proliferation. In addition, the cation exchange ability of bentonite can release charged ions from the bacterial cell wall and disrupt their structure and performance [46]. These properties mean that bentonite has potential uses in a variety of antimicrobial applications, as it could be used in wound dressings, food packaging, and water purification systems [47]. In addition to its direct antimicrobial activity, bentonite has been shown to increase the effectiveness of antibiotics and other antimicrobial agents by adsorbing them to its surface to prevent their degradation.

In laboratory studies, bentonite containing silver nanoparticles was shown to be effective against a variety of pathogens, including *Escherichia coli*, *Salmonella*, *Staphylococcus aureus*, and *Bacillus subtitles* [48,49]. It was found that bentonite has the remarkable ability to absorb coliphages T1 and T7 from *Escherichia coli* when tested in vitro [50]. By combining the clay with water in a ratio of 2–4 parts water to 1 part clay and incubating it with live bacteria at 37 °C for 24 h, the clay exhibited remarkable broad-spectrum antibacterial properties, successfully eliminating the bacterial strains [51]. Moreover, experiments with modified bentonite have also shown promising antibacterial effects [52,53]. These effects are thought to be caused by physical interactions, such as cell penetration or rupture, as well as chemical interactions in which the clay may poison the bacteria or cause nutrient shortages [12]. Moreover, it exhibited antiviral activity against several viruses, including the human immunodeficiency virus (HIV) and the hepatitis B virus (HBV). Bentonite clay also showed significant antifungal activity against *Aspergillus niger* and *Rizopus oryzae* by altering the shape of the cell membrane and preventing proper budding due to membrane integrity degradation [54].

Overall, the antimicrobial activity of bentonite has potential applications in various fields, including food preservation, water purification, and the development of new antimicrobial agents. However, further research is needed to fully understand the mechanisms behind its activity and to determine its potential as an effective and safe alternative to conventional antibiotics.

### 3.2. Side Effects and Adverse Effects

Geophagia, the intentional consumption of soil, is a widespread practice found in both impoverished and industrialized regions globally. While this practice has certain advantages, such as acting as oral and topical antimicrobials, as well as a detoxifier, it can also have negative health effects, particularly if the clay contains impurities [55]. However, bentonite is generally considered to be a safe substance, but its use in human therapy can have some adverse effects, especially when ingested or inhaled in large quantities. Some of the potential adverse effects of bentonite include the following:

*Gastrointestinal distress*: The ingestion of large quantities of clay substances like bentonite can result in the gastrointestinal binding of essential electrolytes and the possible obstruction of the gastrointestinal tract, which causes nausea, constipation, and other unusual feelings [56].

*Respiratory problems*: Silica, primarily found in the form of quartz, poses significant risks due to its sharp grains, potentially leading to severe consequences like bleeding and pulmonary fibrosis [57]. The toxicity of bentonite is heavily dependent on its crystalline silica content. Some bentonites may contain variable amounts of respirable crystalline silica. Bentonite dust and impurities (various forms of crystalline silica) can cause respiratory irritation and can be harmful when inhaled. The prolonged inhalation of bentonite dust can result in lung damage and other respiratory problems [58].

*Skin irritation*: Bentonite can cause skin irritation and itching when applied topically. In some cases, this may result in redness, swelling, itching, and mild eye irritation [58].

*Allergic reactions*: Some modifications of bentonite clay may include substances that can potentially trigger allergic reactions in certain individuals [59].

*Interference with medications*: Calcium-rich clay’s (bentonite) absorbent properties can interfere with the absorption and effectiveness of certain medications, particularly those that are taken orally [34].

*Genotoxicity*: The genotoxicity of natural bentonite and bentonite modified with different concentrations of silica has been investigated. The results revealed that an organo-modified montmorillonite exhibited genotoxicity, suggesting that the observed genotoxic effects were due to the organo-modifier [60]. Similarly, with organic bentonite, genotoxicity could be caused by the particle fraction [61]. Overall, it was found that the genotoxicity potential of bentonite particles is generally low but may be influenced by the quartz content and the presence of transition metals [62].

It is important to note that these negative consequences are usually rare occurrences and tend to happen when bentonite is taken in large doses or not used according to recommended guidelines. Bentonite is considered to be safe when used in recommended amounts and in the manner intended.

Overall, to ensure the safe use of bentonite in human therapy, it is crucial to seek guidance from a healthcare professional. Additionally, it is essential for individuals to understand the potential adverse effects and to carefully follow recommended dosages and usage instructions.

## 4. Bentonite Is a Popular Ingredient in the Cosmetics Industry

Bentonite is well known for its unique properties and is widely used in the cosmetics industry, in which it plays an important role. Here are some examples of bentonite being applied as an ingredient in the field of cosmetics:

*Absorbent*: Due to its excellent absorbent properties, it is a popular ingredient in skin care products such as body washes and face masks, which effectively remove impurities and excess oil to smooth the skin [11]. For instance, activated bentonite was added to electrospun nanofibers to create a product with skin-protective qualities [63]. A mineral-based sunscreen containing activated clay in combination with a dispersing agent and one or more inorganic sunscreens has been patented, representing the development of a mineral sunscreen that offers exceptional spreadability, non-whitening effects, and strong UVB/UVA protection [64].

*Thickening agent*: Bentonite acts as a natural thickener in cosmetic formulations such as lotions and creams, providing a creamy texture without requiring synthetic thickeners [65].

*Clarifying agent*: The clarifying qualities of clay are beneficial in hair care products such as shampoos and conditioners, as they help remove dandruff and soothe the scalp [11].

*Oral care*: In oral care products like toothpaste and mouthwash, the inclusion of bentonite is deemed beneficial due to its ability to eliminate impurities from teeth and gums, along with its potential antibacterial effects [11].

*Deodorant*: When it comes to natural deodorants, the absorbent properties of bentonite make it a suitable choice for combating odor-causing bacteria [11].

*Anti-aging*: More recently, bentonite has been suggested as a cosmetic ingredient with anti-aging properties because it induces telomerase activity (a telomerase activator preserves the length of a telomere and delays the aging process of cells). Cosmetic formulations incorporate bentonite extract to increase telomerase activity to prevent skin aging or enhance anti-aging [66].

There are many companies that manufacture medicines or cosmetics using bentonite as an ingredient. Notably, major global cosmetic and pharmaceutical companies, including L’Oreal, Revlon, Burt’s Bees, Colgate-Palmolive, and Pfizer, integrate bentonite into their formulations [11,67,68,69,70] (Table 1).

These are just a few examples of companies that use bentonite in their products. Many other companies in the pharmaceutical, personal care, and cosmetics industries also use bentonite as an ingredient in their formulations.

Additionally, several Korean companies have incorporated bentonite into their product formulations, as illustrated in Table 2. These companies, such as Amorepacific, Innisfree, LG Household & Health Care, and Kwangdong Pharmaceutical [11,71,72], utilize bentonite across a diverse range of applications, spanning skin care, oral hygiene, and pharmaceuticals.

Beyond these examples, many world-renowned Korean companies have recognized the manifold benefits of bentonite, exploiting its properties in various applications.

Overall, these companies leverage bentonite for purposes ranging from natural thickening and texturizing to its inclusion as an excipient in drug formulations, exemplifying its widespread utilization in the pharmaceutical, personal care, and cosmetics industries.

## 5. Source of Bentonite in South Korea

**Figure 2:**
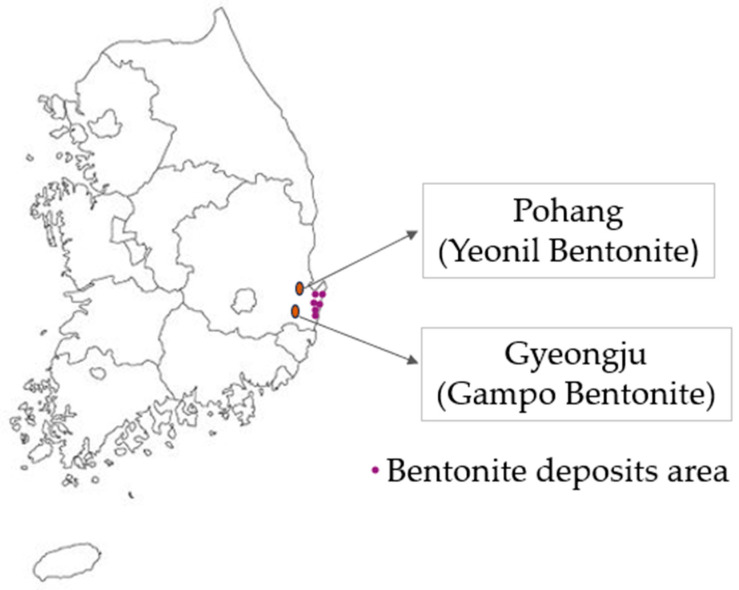
Bentonite deposits in South Korea [76].

In South Korea, Yeonil and Gampo are the representative natural bentonites produced in Pohang and Gyeongju, respectively (Figure 2). Pohang City is home to the largest bentonite deposit in Asia, located in the northern part of the city. The majority of bentonite in these regions is predominantly composed of Ca–montmorillonite [73]. Two types of bentonites are recognized, Na–montmorillonite and Ca–montmorillonite [74,75].

Bentonite has excellent adsorption and ion-exchange properties, which make it useful in a variety of industrial and environmental applications. The bentonite from Pohang City is used in a range of industries, including those related to construction, civil engineering, drilling, and environmental remediation [17]. In particular, the high-quality bentonite from this city is used as a binder in foundry molds, as well as in drilling muds for oil and gas exploration [77]. The mining and processing of bentonite in Pohang City have contributed significantly to the local economy and provided employment opportunities for many residents. However, the environmental impacts of mining activities in this area have been a concern, and efforts are being made to mitigate these impacts through responsible mining practices.

Overall, the bentonite deposits in Pohang City have remarkable economic and industrial value, and the high-quality bentonite from this region is in demand both domestically and internationally.

## 6. Bentonite Research in South Korea

The Korea Institute of Geoscience and Mineral Resources (KIGAM) has conducted extensive research on bentonite, contributing to our understanding of the mineral’s potential applications in various industries. The KIGAM’s research has focused on exploring the properties and characteristics of bentonite, including its adsorption capacity, swelling behavior, and rheological properties [78]. This research has led to advancements in the development of new applications for bentonite, as well as improvements in its processing and utilization techniques. The KIGAM’s work has contributed to the understanding of the geological processes involved in the formation of bentonite deposits, which are crucial for identifying potential sources and optimizing extraction methods [79]. Here are some examples of the KIGAM’s studies on bentonite and functional clays:

*Mineralogical and physicochemical characterization*: The KIGAM has conducted in-depth mineralogical and physicochemical studies of bentonite and other clays, including studies on their chemical compositions, crystal structures, and surface properties. These studies allow us to understand the unique properties of these materials and their potential applications [78].

*Environmental remediation*: The KIGAM has studied the application of bentonite and other clays on environmental remediation, such as the removal of heavy metals from soil and water [80]. These studies have shown that clays can be effective in absorbing and immobilizing contaminants, making them a promising tool for environmental cleanups.

*Geotechnical applications*: The KIGAM has also conducted research on the geotechnical applications of bentonite, including its use in construction, in tunneling, and as a drilling mud. According to these findings, clays can help to regulate water flow and enhance the strength and stability of rock and soil [81].

*Health and biomedical applications*: the KIGAM has been researching the potential use of bentonite and other clays for medical applications in healthcare, such as drug delivery and wound healing. These studies show that clays have unique properties such as high surface areas, good water absorption and release capacities, and antimicrobial properties that are useful in biomedical applications [9].

Overall, the KIGAM’s research on bentonite and activated clays has contributed to a better understanding of the characteristics and potential applications of these materials. Their research has shown that these clays have a wide range of applications in various industries, including the environmental remediation, geotechnical engineering, and healthcare, and biomedicine industries.

## 7. Future Research Directions Regarding Bentonite in Biomedical and Cosmetics Industries

Future research on bentonite in biomedicine and the cosmetics industry could explore multifaceted approaches to advance its applications. In biomedicine, investigations may focus on elucidating the mechanisms of interaction between bentonite and biological systems, particularly in drug delivery systems and tissue engineering. Understanding the biocompatibility, long-term safety, and potential therapeutic benefits of bentonite in diverse biomedical contexts could pave the way for innovative medical interventions. Nevertheless, several potential research directions merit consideration:

*Antidiarrheal*: Both bentonite clay and bacteriophages have shown promise in controlling the symptoms and pathogenicity of bacteria-induced diarrhea. Bentonite clay’s ability to adsorb and neutralize protein toxins of pathogens may help reduce diarrhea, while the specificity of bacteriophages for bacteria provides a targeted approach to eliminating the causative agent [80,82]. Additionally, future research could focus on optimizing delivery systems to enhance the efficacy of this combination, ensuring targeted and sustained release for maximum therapeutic impact.

*Probiotic*: A case report presented the successful treatment of a pediatric clinical case of *C. difficile* with naturopathic and complementary alternative medicines (CAMs) such as black seed oil (*Nigella sativa*), bentonite clay, and probiotics. Within just four days of combining these CAMs, stool immunoassays revealed the complete eradication of the infection. Further research is warranted to determine the effectiveness and suitable doses of black seed oil, bentonite clay, and probiotics in treating CDI (*C. difficile* infection) in children over the course of life [83]. Additionally, bentonite clay could potentially function as an intestine-healing or probiotic-protecting (protecting probiotics from excessively acidic environments) agent. Exfoliated bentonite/alginate nanocomposite hydrogels may be potential vehicles for the delivery of probiotics to the gut. To ensure that probiotics are safely and effectively incorporated into gastrointestinal health strategies, more research is required to completely define the potential advantages and risks related to this delivery method [84].

*Antibacterial*: Bentonite clay has been used internally and externally to treat diseases in traditional medicine for centuries [85]. Clay has antibacterial effects against drug-resistant pathogens without toxic side effects [86]. Bentonite clay has shown a remarkable ability to bind and neutralize bacterial protein toxins, which makes it a valuable treatment for diarrhea and cholera [18,87]. Remarkably, many studies have also highlighted the standalone efficacy of bentonite clay [18,88,89]. The results suggest that bentonite clay may be a useful source of antibacterial agents that require further investigation for therapeutic applications.

*Antiviral*: Bentonite has detoxifying and antiviral properties. For dermal sanitization, bentonite paste exhibits strong interactions with a variety of +RNA viruses, including SARS-CoV-2 and poliovirus. The regular use of bentonite paste to cleanse one’s hands not only offers total protection against viruses but also enhances skin health [90]. Furthermore, the virus-capturing properties of bentonite are a key ingredient in the medical device AM-301 (Bentrio). Its thin aluminum silicate sheets carry a negative charge, allowing it to adsorb viral particles and drugs. AM-301, composed of safe ingredients, exhibits thixotropic properties due to bentonite, making it easy to apply as a nasal spray that forms a protective film on the nasal epithelium. Further investigations are needed in vivo and in humans to evaluate the efficacy of bentonite in a broad spectrum of actions against a range of viruses, allergens, and pollutants [41].

*Antioxidant and antidiabetic*: Focusing on mechanisms such as adsorption and release, bentonite could provide insights into the modulation of antioxidant activity. Studies evaluating the influence of bentonite on the antioxidant capacity in biological systems such as cell cultures or animal models could further elucidate its physiological effects. Moreover, researchers may explore the possible synergistic effects of bentonite in combination with particular antioxidants to produce improved antioxidant formulations [91]. While bentonite clay has shown promise in in vitro studies with respect to lowering blood sugar and mitigating diabetic complications, further research is crucial to solidify our understanding of its antidiabetic potential [92]. Future directions should focus on elucidating the precise mechanisms behind these effects, conducting robust in vivo trials to validate efficacy and safety, exploring targeted delivery systems for enhanced bioavailability, investigating potential synergistic effects with other antioxidant and antidiabetic agents, and prioritizing comprehensive toxicity and long-term safety assessments.

Concurrently, in the cosmetics industry, researchers might focus on optimizing formulations for skin care products, exploring new combinations with natural components, and evaluating the efficacy of bentonite in personalized cosmetic applications [93]. Investigations of its role in enhancing product stability, texture, and skin-related benefits such as absorption and detoxification may contribute to the development of novel cosmetic products [11]. Future studies may potentially focus on the development of smart cosmetic formulations incorporating bentonite, integrating technologies like controlled-release systems for prolonged benefits. As the cosmetics industry continuously grows, these research directions have the potential to open up new avenues for the sustainable and innovative use of bentonite in cosmetic applications.

Overall, by addressing aspects related to both biomedical and cosmetics applications, future research may provide a holistic knowledge of the potential of bentonite across diverse industries, resulting in significant insights for practical applications and further innovations.

## 8. Conclusions

Bentonite, an abundant natural element in parts of South Korea, is useful in various biomedical and cosmetics applications. This exploration of its geological abundance and mineralogical composition highlighted the unique properties that position Korean bentonite as a versatile material with immense potential. As we anticipate their further exploration through research endeavors, we also anticipate that both natural and modified organic bentonite will play significant roles in advancing the biomedical and cosmetics industries. In the biomedical arena, the mineral’s role in drug delivery systems and therapeutic formulations emerges as a promising area for further investigation. Simultaneously, the cosmetics industry stands to benefit from the mineral’s application in skin care formulations, reflecting a convergence of scientific innovation and consumer-driven demands.

The integration of locally sourced bentonite into research initiatives is not merely an academic pursuit, but a strategic imperative for industries seeking eco-friendly and regionally tailored solutions. This review emphasized the importance of harmonizing scientific research with industrial applications, providing a roadmap for utilizing the unique properties of bentonite to meet the evolving demands of these sectors. In doing so, we aspire to usher in a new era where bentonite from Korea becomes a vital component of cutting-edge innovations in both the biomedical and cosmetics industries.

## Figures and Tables

**Table 1:** Utilization of bentonite by different companies in personal care and healthcare products.

Company	Reagent	Usages	Function	Reference
L’Oreal	Face masks	Skin care products	To help absorb excess oil and impurities from the skin	[11]
Revlon	Nanocosmetics	Lipstick and Nail polish products	Have unique rheological properties, ensure improved application on the skin, dry quickly, enhance adherence, and are easy to remove	[67]
Burt’s Bees	Detoxifying Clay Mask	Skin care products	To help draw out impurities and purify the skin	[68]
Colgate-Palmolive	Toothpaste	Oral care products	To help prevent plaque and tartar buildup on teeth	[69]
Pfizer	Tablets	Drug formulations	As an excipient, helps to bind the active ingredients and provide a smooth tablet surface	[70]

**Table 2:** Bentonite in cosmetics and pharmaceuticals in South Korea.

Company	Reagent	Usages	Function	Reference
Amorepacific	Cosmetics	Skin care products: clay mask	To help absorb impurities and control sebum	[11]
Innisfree	Cosmetics	Skin care products: volcanic clay mask	To help draw out impurities and excess oil from the skin	[71]
LG Household & Health Care	Toothpaste and cosmetics	Toothpaste, rip rouge	To help remove plaque	[11]
Kwangdong Pharmaceutical	Pharmaceuticals	Nanohybrid coated with acid-soluble polymer	To enhance both taste masking and the rapid drug delivery	[72]

## References

[1] Christidis G.E., Huff W.D. (2009). Geological Aspects and Genesis of Bentonites. Elements.

[2] Bell K., Parker S. (1988). McGraw-Hill Encyclopedia of the Geological Sciences.

[3] Odom I.E. (1984). Smectite clay minerals: Properties and uses. Philos. Trans. R. Soc. Lond. Ser. A Math. Phys. Sci..

[4] Eisenhour D.D., Brown R.K. (2009). Bentonite and Its Impact on Modern Life. Elements.

[5] Mi J.Z., Gregorich E.G., Xu S.T., McLaughlin N.B., Ma B., Liu J.H. (2021). Changes in soil biochemical properties following application of bentonite as a soil amendment. Eur. J. Soil Biol..

[6] Soliemanzadeh A., Fekri M. (2017). The application of green tea extract to prepare bentonite-supported nanoscale zero-valent iron and its performance on removal of Cr(VI): Effect of relative parameters and soil experiments. Microporous Mesoporous Mater..

[7] Gens A., Guimaraes L.D.N., Garcia-Molina A., Alonso E.E. (2002). Factors controlling rock–clay buffer interaction in a radioactive waste repository. J. Eng. Geol..

[8] Dardir F.M., Mohamed A.S., Abukhadra M.R., Ahmed E.A., Soliman M.F. (2018). Cosmetic and pharmaceutical qualifications of Egyptian bentonite and its suitability as drug carrier for Praziquantel drug. Eur. J. Pharm. Sci..

[9] Park J.H., Shin H.J., Kim M.H., Kim J.S., Kang N., Lee J.Y., Kim K.T., Lee J.I., Kim D.D. (2016). Application of montmorillonite in bentonite as a pharmaceutical excipient in drug delivery systems. J. Pharm. Investig..

[10] Gamoudi S., Srasra E. (2017). Characterization of Tunisian clay suitable for pharmaceutical and cosmetic applications. Appl. Clay Sci..

[11] Viseras C., Sánchez-Espejo R., Palumbo R., Liccardi N., García-Villén F., Borrego-Sánchez A., Massaro M., Riela S., López-Galindo A. (2021). Clays in cosmetics and personal-care products. Clays Clay Miner..

[12] Williams L.B., Haydel S.E., Ferrell R.E. (2009). Bentonite, bandaids, and borborygmi. Elements.

[13] Damato A., Vianello F., Novelli E., Balzan S., Gianesella M., Giaretta E., Gabai G. (2022). Comprehensive review on the interactions of clay minerals with animal physiology and production. Front. Vet. Sci..

[14] Deng Z., Jang K.B., Jalukar S., Du X., Kim S.W. (2023). Efficacy of Feed Additive Containing Bentonite and Enzymatically Hydrolyzed Yeast on Intestinal Health and Growth of Newly Weaned Pigs under Chronic Dietary Challenges of Fumonisin and Aflatoxin. Toxins.

[15] Diamond J.M. (1999). Evolutionary biology. Dirty eating for healthy living. Nature.

[16] Kreulen D.A. (1985). Lick Use by Large Herbivores—A Review of Benefits and Banes of Soil Consumption. Mamm. Rev..

[17] Kim S.O., Wang S. (2023). Behavior of Heavy Metals Studies on the Hydrothermal Alteration Characteristics of Bentonite; Use as Medicinal Mineral. Econ. Environ. Geol..

[18] Damrau F. (1961). The value of bentonite for diarrhea. Med. Ann. Dist. Columbia.

[19] Kim G.J., Kim D., Lee K.J., Kim D., Chung K.H., Choi J.W., An J.H. (2020). Effect of Nano-Montmorillonite on Osteoblast Differentiation, Mineral Density, and Osteoclast Differentiation in Bone Formation. Nanomaterials.

[20] Roh K.-M. *Technical Development for Practicalizing Medical Clays*; M. o. S. a. ICT: 2019. https://www.law.go.kr/%ED%96%89%EC%A0%95%EA%B7%9C%EC%B9%99/%EB%8C%80%ED%95%9C%EB%AF%BC%EA%B5%AD%EC%95%BD%EC%A0%84.

[21] Jin S.E., Lee J.I., Hwang S.J. (2015). Case Study of Pharmaceutical Ingredients Derived from Clay Minerals. Econ. Environ. Geol..

[22] Pohl P., Bielawska-Pohl A., Dzimitrowicz A., Jamroz P., Welna M. (2018). Impact and practicability of recently introduced requirements on elemental impurities. TrAC Trends Anal. Chem..

[23] Mahmoodi N.M., Taghizadeh A., Taghizadeh M., Baglou M.A.S. (2019). Surface modified montmorillonite with cationic surfactants: Preparation, characterization, and dye adsorption from aqueous solution. J. Environ. Chem. Eng..

[24] Zakaria R.M., Hassan I., El-Abd M.Z., El-Tawil Y.A. Lactic acid removal from wastewater by using different types of activated clay. Proceedings of the Thirteenth International Water Technology Conference (IWTC).

[25] Rethinasabapathy M., Kang S.M., Lee I., Lee G.W., Hwang S.K., Roh C., Huh Y.S. (2018). Layer-Structured POSS-Modified Fe-Aminoclay/Carboxymethyl Cellulose Composite as a Superior Adsorbent for the Removal of Radioactive Cesium and Cationic Dyes. Ind. Eng. Chem. Res..

[26] Nadziakiewicza M., Kehoe S., Micek P. (2019). Physico-Chemical Properties of Clay Minerals and Their Use as a Health Promoting Feed Additive. Animals.

[27] Ramos A.J., FinkGremmels J., Hernandez E. (1996). Prevention of toxic effects of mycotoxins by means of nonnutritive adsorbent compounds. J. Food Prot..

[28] Gomez M.D.M., Brandt R., Jakubowski N., Andersson J.T. (2004). Changes of the metal composition in German white wines through the winemaking process. A study of 63 elements by inductively coupled plasma-mass spectrometry. J. Agric. Food Chem..

[29] Liu J., Zhao L., Liu Q., Li J., Qiao Z., Sun P., Yang Y. (2022). A critical review on soil washing during soil remediation for heavy metals and organic pollutants. Int. J. Environ. Sci. Technol..

[30] Singh G., Ramadass K., Sooriyakumar P., Hettithanthri O., Vithange M., Bolan N., Tavakkoli E., Van Zwieten L., Vinu A. (2022). Nanoporous materials for pesticide formulation and delivery in the agricultural sector. J. Control. Release.

[31] Carretero M.I., Pozo M. (2010). Clay and non-clay minerals in the pharmaceutical and cosmetic industries Part II. Active ingredients. Appl. Clay Sci..

[32] Kalasz H., Antal I. (2006). Drug Excipients. Curr. Med. Chem..

[33] Viseras C., Cerezo P., Sanchez R., Salcedo I., Aguzzi C. (2010). Current challenges in clay minerals for drug delivery. Appl. Clay Sci..

[34] Carretero M.I., Pozo M. (2009). Clay and non-clay minerals in the pharmaceutical industry Part I. Excipients and medical applications. Appl. Clay Sci..

[35] Bonina F.P., Giannossi M.L., Medici L., Puglia C., Summa V., Tateo F. (2007). Adsorption of salicylic acid on bentonite and kaolin and release experiments. Appl. Clay Sci..

[36] Alkrad J.A., Abu Shmeis R., Alshwabkeh I., Abazid H., Mohammad M.A. (2017). Investigation of the potential application of sodium bentonite as an excipient in formulation of sustained release tablets. Asian J. Pharm..

[37] Jawahar S., Nafar A., Paray B.A., Al-Sadoon M.K., Balasundaram C., Harikrishnan R. (2018). Bentonite clay supplemented diet on immunity in stinging catfish, *Heteropneustes fossilis* against *Aeromonas hydrophila*. Fish Shellfish Immunol..

[38] Viseras C., Aguzzi C., Cerezo P., Lopez-Galindo A. (2007). Uses of clay minerals in semisolid health care and therapeutic products. Appl. Clay Sci..

[39] Gora W., Gora P., Jaszczyszyn K. (2016). Perspectives of natural bentonite application in industrial wastewater treatment. Rocz. Ochr. Srodowiska.

[40] Suman J.D. (2013). Current understanding of nasal morphology and physiology as a drug delivery target. Drug Deliv. Transl. Res..

[41] Fais F., Juskeviciene R., Francardo V., Mateos S., Guyard M., Viollet C., Constant S., Borelli M., Hohenfeld I.P. (2022). Drug-free nasal spray as a barrier against SARS-CoV-2 and its delta variant: In vitro study of safety and efficacy in human nasal airway epithelia. Int. J. Mol. Sci..

[42] Cabuk M., Alan Y., Unal H.I. (2017). Enhanced electrokinetic properties and antimicrobial activities of biodegradable chitosan/organo-bentonite composites. Carbohydr. Polym..

[43] Pouraboulghasem H., Ghorbanpour M., Shayegh R., Lotfiman S. (2016). Synthesis, characterization and antimicrobial activity of alkaline ion-exchanged ZnO/bentonite nanocomposites. J. Cent. South Univ..

[44] Luukkonen T., Bhuyan M., Hokajarvi A.M., Pitkanen T., Miettinen I.T. (2022). Water disinfection with geopolymer-bentonite composite foam containing silver nanoparticles. Mater. Lett..

[45] Meschke J.S., Sobsey M.D. (1998). Comparative adsorption of Norwalk virus, poliovirus 1 and F+ RNA coliphage MS2 to soils suspended in treated wastewater. Water Sci. Technol..

[46] Williams L.B., Haydel S.E. (2010). Evaluation of the medicinal use of clay minerals as antibacterial agents. Int. Geol. Rev..

[47] Mokhtari-Hosseini Z.B., Hatamian-Zarmi A., Mahdizadeh S., Ebrahimi-Hosseinzadeh B., Alvandi H., Kianirad S. (2022). Environmentally-Friendly Synthesis of Ag Nanoparticles by Fusarium sporotrichioides for the Production of PVA/Bentonite/Ag Composite Nanofibers. J. Polym. Environ..

[48] Motshekga S.C., Ray S.S., Onyango M.S., Momba M.N.B. (2013). Microwave-assisted synthesis, characterization and antibacterial activity of Ag/ZnO nanoparticles supported bentonite clay. J. Hazard. Mater..

[49] Santos M.F., Oliveira C.M., Tachinski C.T., Fernandes M.P., Pich C.T., Angioletto E., Riella H.G., Fiori M.A. (2011). Bactericidal properties of bentonite treated with Ag plus and acid. Int. J. Miner. Process..

[50] Schiffenbauer M., Stotzky G. (1982). Adsorption of coliphages T1 and T7 to clay minerals. Appl. Environ. Microbiol..

[51] Haydel S.E., Remenih C.M., Williams L.B. (2008). Broad-spectrum in vitro antibacterial activities of clay minerals against antibiotic-susceptible and antibiotic-resistant bacterial pathogens. J. Antimicrob. Chemother..

[52] Plachá D., Rosenbergová K., Slabotínský J., Kutláková K.M., Študentová S., Martynková G.S. (2014). Modified clay minerals efficiency against chemical and biological warfare agents for civil human protection. J. Hazard. Mater..

[53] Shameli K., Ahmad M.B., Zargar M., Yunus W.M.Z.W., Ibrahim N.A., Shabanzadeh P., Moghaddam M.G. (2011). Synthesis and characterization of silver/montmorillonite/chitosan bionanocomposites by chemical reduction method and their antibacterial activity. Int. J. Nanomed..

[54] Khan M.M., Bhatti Q.A., Akhlaq M., Ishaq M., Ali D., Jalil A., Asghar J., Alarifi S., Elaissari A. (2022). Assessment of Antimicrobial Potential of Plagiochasma rupestre Coupled with Healing Clay Bentonite and AGNPS. BioMed Res. Int..

[55] Maxim L.D., Niebo R., McConnell E.E. (2016). Bentonite toxicology and epidemiology—A review. Inhal. Toxicol..

[56] Bennett A., Stryjewski G. (2006). Severe hypokalemia caused by oral and rectal administration of bentonite in a pediatric patient. Pediatr. Emerg. Care.

[57] Horwell C.J., Baxter P.J. (2006). The respiratory health hazards of volcanic ash: A review for volcanic risk mitigation. Bull. Volcanol..

[58] Maxim L.D., Niebo R., McConnell E.E. (2014). Perlite toxicology and epidemiology—A review. Inhal. Toxicol..

[59] Bangar S.P., Ilyas R.A., Chowdhury A., Navaf M., Sunooj K.V., Siroha A.K. (2023). Bentonite clay as a nanofiller for food packaging applications. Trends Food Sci. Technol..

[60] Sharma A.K., Schmidt B., Frandsen H., Jacobsen N.R., Larsen E.H., Binderup M.L. (2010). Genotoxicity of unmodified and organo-modified montmorillonite. Mutat. Res..

[61] Li X.X., Zhang M.B., Lu Y.Z., Yan S.X., Chen Q., Xing M.L., Zou H., Chen S.J., and He J.L. (2010). Genotoxicity of organic bentonite particles in vitro. Chinese Journal of Industrial Hygiene and Occupational Diseases. Zhonghua Lao Dong Wei Sheng Zhi Ye Bing Za Zhi.

[62] Geh S., Shi T., Shokouhi B., Schins R.P., Armbruster L., Rettenmeier A.W., Dopp E. (2006). Genotoxic potential of respirable bentonite particles with different quartz contents and chemical modifications in human lung fibroblasts. Inhal. Toxicol..

[63] Bazbouz M.B., Russell S.J. (2018). Cellulose acetate/sodium-activated natural bentonite clay nanofibres produced by free surface electrospinning. J. Mater. Sci..

[64] Timothy G.R.A.Y., Cziryak P., Kljuic A. (2015). Mineral Sunscreen Composition and Process for Protecting Skin from Photodamage and Aging. U.S. Patent.

[65] Babahoum N., Ould Hamou M. (2021). Characterization and purification of Algerian natural bentonite for pharmaceutical and cosmetic applications. BMC Chem..

[66] Bae K.B., Choi B.H., Jang Y.J., Kim D.Y., Lee E.J. (2021). Cosmetic Composition for Preventing or Improving Skin Anti-Aging Containing Bentonite Extract for Increasing Telomerase Activity. (K. R. Patent No. 102228930B1).

[67] Moraes C.A.P., Vieira A.R. (2020). Nanomaterials for lip and nail cares applications. Nanocosmetics.

[68] Grigale-Soročina Z., Birks I., Kalniņš M. (2017). Processing Technology Development for Organic Clay Suspension System. Solid State Phenom..

[69] Greenwall L.H., Greenwall-Cohen J., Wilson N.H. (2019). Charcoal-containing dentifrices. Br. Dent. J..

[70] Lee J.H., Choi G., Oh Y.J., Park J.W., Choy Y.B., Park M.C., Choy J.H. (2012). A nanohybrid system for taste masking of sildenafil. Int. J. Nanomed..

[71] Nguyen J.K., Masub N., Jagdeo J. (2020). Bioactive ingredients in Korean cosmeceuticals: Trends and research evidence. Cosmet. Dermatol..

[72] Gosavi D.S., Akarte A.M., Chaudhari P.M., Wagh K.S., Patil P.H. (2021). Mouth dissolving films: A review. World J. Pharm. Res..

[73] Kim M., Lee S., Cheon E., Kim M., Yoon S. (2021). Thermochemical changes on swelling pressure of compacted bentonite. Ann. Nucl. Energy.

[74] Gray M.N., Cheung S.C.H., Dixon D.A. (1984). The Influence of Sand Content on Swelling Pressures and Structure Developed in Statically Compacted Na-Bentonite (No. AECL--7825).

[75] Villar M.V., Rivas P. (1994). Hydraulic properties of montmorillonite-quartz and saponite-quartz mixtures. Appl. Clay Sci..

[76] Hong S., Kim J., Um W. (2022). Surface Modification of Bentonite for the Improvement of Radionuclide Sorption. J. Nucl. Fuel Cycle Waste Technol..

[77] Park S.S., Doan N.P., Jeong S.W. (2020). Numerical simulation of water content dependent undrained shear strength of clays. Mar. Georesour. Geotechnol..

[78] Kong M., Lee M., Kim G.Y., Jang J., Kim J.S. (2023). Characterization of Compacted Ca- and Na-Bentonite with Copper Corrosion Products in the KAERI Underground Research Tunnel. Minerals.

[79] Kim C., Kim J.H., Chwae U. (2006). Geophysical Investigations of Quaternary Fault in the Southeastern Coast of Korean Peninsula. Proceedings of the 8th SEGJ International Symposium.

[80] Fiorentin L., Vieira N.D., Barioni W., Cruz C. (2005). Detection and in vitro antibiotic sensitivity of *salmonella* spp. isolated from healthy and diarrheic piglets in southern Brazil. Pesqui Vet. Bras..

[81] Koh S.M., Song M.S. (2008). Mineralogy and physicochemistry of smectites saturated with inorganic and organic cations. Clay Sci..

[82] Lusiastuti A.M., Tabbu C.R., Kusdiyantini E., Sediawan W.B. (2019). Utilization of clay-iron nanoparticle composites in combination with biosurfactants as antibacterial agents against *Salmonella Typhimurium*. Molecules.

[83] Littman E., Winningham N., Carson T.B., Hidalgo I.M. (2022). Black Seed Oil, Bentonite Clay, and Probiotics: A Comprehensive Holistic Cure for *Clostridium difficile* Infection in a 2-Year-Old Female Child. Case Rep. Infect. Dis..

[84] Kim J., Hlaing S.P., Lee J., Saparbayeva A., Kim S., Hwang D.S., Lee E.H., Yoon I.S., Yun H., Kim M.S. (2021). Exfoliated bentonite/alginate nanocomposite hydrogel enhances intestinal delivery of probiotics by resistance to gastric pH and on-demand disintegration. Carbohydr. Polym..

[85] Moosavi M. (2017). Bentonite clay as a natural remedy: A brief review. Iran. J. Public Health.

[86] Behroozian S., Svensson S.L., Davies J. (2016). Kisameet clay exhibits potent antibacterial activity against the ESKAPE pathogens. MBio.

[87] Sturino J.M., Pokusaeva K., Carpenter R. (2015). Effective sequestration of Clostridium difficile protein toxins by calcium aluminosilicate. Antimicrob. Agents Chemother..

[88] Gilbert B., Lienhardt A., Palomera S., Barberis L., Borreda D. (1991). The efficacy of smectite in acute infantile diarrhea, compared to a placebo and loperamide. Ann. Pediatr..

[89] Pérez-Gaxiola G., Cuello-García C.A., Florez I.D., Pérez-Pico V.M. (2018). Smectite for acute infectious diarrhoea in children. Cochrane Database Syst. Rev..

[90] Das P., Tadikonda B.V. (2020). Bentonite clay: A potential natural sanitizer for preventing neurological disorders. ACS Chem. Neurosci..

[91] Hernández D., Montalvo A., Pérez I., Charnay C., Sánchez-Espejo R., Cerezo P., Viseras C., Riela S., Cinà G., Rivera A. (2023). Antioxidant Efficacy and “In Vivo” Safety of a Bentonite/Vitamin C Hybrid. Pharmaceutics.

[92] Rudayni H.A., Shemy M.H., Aladwani M., Alneghery L.M., Abu-Taweel G.M., Allam A.A., Abukhadra M.R., Bellucci S. (2023). Synthesis and Biological Activity Evaluations of Green ZnO-Decorated Acid-Activated Bentonite-Mediated Curcumin Extract (ZnO@ CU/BE) as Antioxidant and Antidiabetic Agents. J. Funct. Biomater..

[93] da Silva Favero J., dos Santos V., Weiss-Angeli V., Gomes L.B., Veras D.G., Dani N., Mexias A.S., Bergmann C.P. (2019). Evaluation and characterization of Melo Bentonite clay for cosmetic applications. Appl. Clay Sci..

